# Choroidal Patterns in Stargardt Disease: Correlations with Visual Acuity and Disease Progression

**DOI:** 10.3390/jcm8091388

**Published:** 2019-09-05

**Authors:** Alessandro Arrigo, Alessio Grazioli, Francesco Romano, Emanuela Aragona, Alessandro Bordato, Carlo di Nunzio, Andrea Sperti, Francesco Bandello, Maurizio Battaglia Parodi

**Affiliations:** 1 Department of Ophthalmology, IRCCS San Raffaele Hospital, Vita-Salute University, 20123 Milan, Italy (A.G.) (F.R.) (E.A.) (A.B.) (C.d.N.) (A.S.) (F.B.) (M.B.P.); 2 Eye Clinic, Department of Biomedical and Clinical Science, Luigi Sacco University Hospital, 20100 Milan, Italy

**Keywords:** Stargardt disease, OCT, OCTA, choroid, Sattler layer, Haller layer

## Abstract

Background: To identify different choroidal patterns in Stargardt disease (STGD) and to assess their clinical correlates. Methods: 100 STGD eyes (29 males; mean age 42.6 ± 16.5 years) and 100 control eyes (29 males; mean age 43.2 ± 8.5 years) were included. Optical coherence tomography (OCT) and OCT angiography (OCTA) images were obtained. Four different choroidal patterns, quantitative OCT and OCTA parameters were assessed and statistically analyzed. The main outcome was the correlation between each choroidal pattern and anatomical and functional retinal status. Furthermore, we assessed structural and best corrected visual acuity (BCVA) changes of each STGD subgroup after one-year. Results: Mean BCVA was 0.63 ± 0.44 LogMAR for STGD patients and 0.0 ± 0.0 LogMAR for controls (*p* < 0.01). All quantitative parameters appeared deteriorated in STGD compared to controls (*p* < 0.01). Choroidal patterns were distributed as follows: Pattern 1 (normal appearing choroid) (15%), Pattern 2 (reduced Sattler or Haller layer) (29%), Pattern 3 (reduced Sattler and Haller layers) (26%), Pattern 4 (Pattern 3 + choroidal caverns) (30%). More advanced patterns significantly correlated with a more severe loss of retinal structural integrity. Furthermore, only Pattern 3 and Pattern 4 showed remarkable signs of progression after one year. Conclusions: Choroidal patterns were related with retinal structural status and BCVA loss, and with different disease progression.

## 1. Introduction

Stargardt disease (STGD) is the most common retinal hereditary dystrophy arising at a young age, affecting about 10 to 12.5 individuals per 100,000 in the USA [1]. Transmission is prevalently autosomal recessive, with more than 800 different variants mapped along the *ABCA4* gene [2].

STGD is considered highly heterogeneous in terms of retinal involvement; indeed, the disease’s phenotypes may vary among patients from mild forms to extremely aggressive ones [2,3]. Extensive evidence highlighted the involvement of the choriocapillaris/choroidal complex as a key factor for the onset and progression of retinal atrophy [4,5]. In particular, multimodal imaging revealed a markedly reduced choroidal vascularity index in STGD, together with a wide loss of the choriocapillaris, with complete exposure of underlying choroidal vessels. However, deep investigations of choroidal and choriocapillaris involvement in STGD is still missing.

The aim of this study was to identify and classify different choroidal patterns in STGD and to evaluate the possible correlations with BCVA and peripheral involvement. Moreover, our study was focused on the assessment of the relationship between the choroidal patterns identified and the multimodal imaging quantitative parameters, namely retinal thickness and OCTA parameters, as well as to assess progression markers after one year of follow-up.

## 2. Methods

The study was designed as a prospective, observational case series with one-year of follow-up. All the patients were recruited in the Ophthalmology Unit of San Raffaele Hospital (Milan, Italy) from August 2016 to December 2017. All patients with genetically confirmed diagnosis of STGD signed an informed consent; the study was conducted in accordance with the Declaration of Helsinki and it was approved by the Ethical Committee of the Vita-Salute San Raffaele University in Milan.

The inclusion criteria were: clinical and genetic diagnosis of STGD, age >18 years. The exclusion criteria included: high media opacity, any other type of retinal or optic nerve diseases, ophthalmologic surgery within the previous three months, refractive error > ± 3D, any systemic condition potentially affecting the analyses.

Complete ophthalmologic examination included the following assessments: best corrected visual acuity (BCVA) measurement using standard ETDRS charts, slit-lamp biomicroscopy of anterior and posterior segments, Goldmann applanation tonometry. Fundus autofluorescence and structural OCT images were acquired by means of Spectralis HRA + OCT (Heidelberg Engineering; Heidelberg, Germany). Structural OCT acquisition protocol included high-resolution (ART > 30) raster, radial and dense scans with enhanced depth imaging (EDI). An age- and sex-matched control group was considered for clinical and imaging comparisons.

The following parameters were extracted from the structural OCT: central macular thickness (CMT), retinal nerve fiber layer (RNFL), ganglion cell layer (GCL), inner plexiform layer (IPL), inner nuclear layer (INL), outer plexiform layer (OPL), outer nuclear layer (ONL) and ellipsoid zone-retinal pigment epithelium layer (EZ-RPE). Retinal and choroidal hyperreflective foci (HF) numbers were also calculated. In detail, a vertical line passing through the foveal center was traced on the horizontal structural OCT. From this line, we traced two horizontal 750-μm lines, respectively oriented on the left and the right, to delimit the region of interest for the calculation.

OCTA images were obtained using a swept source OCT DRI Topcon Triton (Topcon Corporation, Tokyo, Japan). OCTA scans included high-resolution 3 × 3 mm and 4.5 × 4.5 mm acquisitions. Only high-quality images (Topcon Imaging Quality factor > 70) were considered.

Superficial (SCP), deep (DCP) and choriocapillary (CC) plexa were automatically segmented and carefully inspected by an expert ophthalmologist (MBP). All reconstructions were loaded in ImageJ software (https://imagej.net/Welcome). In-house scripts were built in order to calculate the following parameters: vessel density (VD), vessel tortuosity (VT), vessel dispersion (VDisp) and vessel rarefaction (VR), as previously described [4]. The foveal avascular zone (FAZ) was manually segmented and excluded from the calculations. We identified and assigned patients to four qualitatively and quantitatively different choroidal patterns by means of structural OCT: Pattern 1 (normal appearing choroid), Pattern 2 (reduced Sattler or Haller layer), Pattern 3 (reduced Sattler and Haller layers), and Pattern 4 (reduced Sattler and Haller layers with choroidal caverns). In particular, the quantitative evaluation included the measures of Sattler and Haller layers on a high-resolution, horizontal EDI structural OCT scan. For all choroidal measures, namely choroidal total thickness, and Sattler and Haller layers thicknesses, we performed three different samplings: one subfoveal and two on the left and right sides, respectively, placed at 750 μm from the fovea. The mean value of the three samplings was considered for the statistical analysis.

The primary outcome was the identification of different choroidal patterns in STGD. Secondary outcomes were the correlations assessment with imaging and functional parameters and the analysis of the disease’s progression over a one-year follow-up.

The statistical analyses were performed using the one-way ANOVA test with Bonferroni correction for multiple comparisons (SPSS, Chicago, IL, USA), with statistical significance set at *p* < 0.05.

Tau-Kendall correlation analysis was used to assess the statistical relationships among the following parameters: BCVA, CMT, CT, VD, VT, VR and VDisp.

## 3. Results

Overall, fifty-four consecutive patients with clinically and genetically confirmed diagnosis of STGD were recruited. One patient was excluded for high media opacities and three patients because of systemic conditions potentially affecting the results of the study (two with uncontrolled arterial hypertension and one with diabetes mellitus).

One-hundred eyes (50 patients) were included in our analysis (29 males; mean age 42.6 ± 16.5 years). Moreover, 100 eyes of healthy control subjects (29 males; mean age 43.2 ± 8.5 years) were also included. Mean BCVA was of 0.63 ± 0.44 LogMAR for STGD group and 0.0 ± 0.0 LogMAR for controls (*p* < 0.01). All clinical and imaging findings are reported in Table 1.

**Figure 1:**
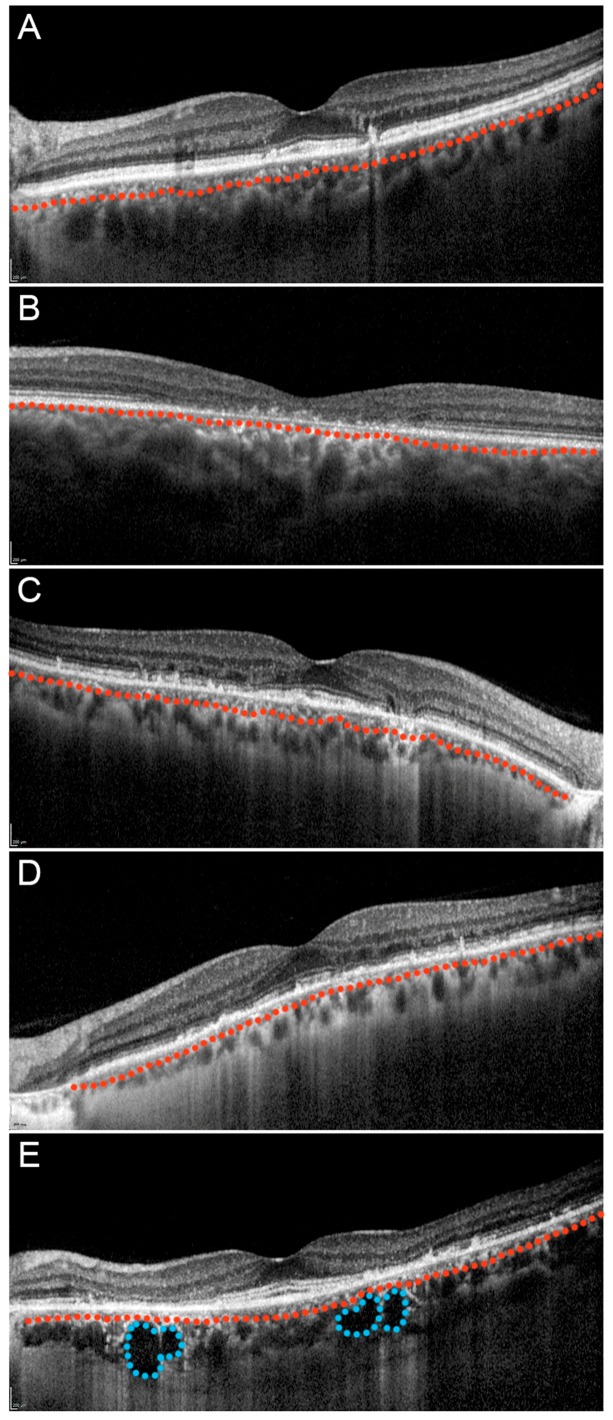
Choroidal patterns in Stargardt disease. **A**: Pattern 1 with a normal appearing choroid; **B**: Pattern 2 with a reduced Sattler layer; **C**: Pattern 2 with a reduced Haller layer; **D**: Pattern 3 with both reduced Sattler and Haller layers; E: Pattern 4 with reduced Sattler and Haller layers with choroidal caverns. In all images, Sattler and Haller layers are separated by a red dotted line. Moreover, choroidal cavers are highlighted by blue dotted lines.

**Figure 2:**
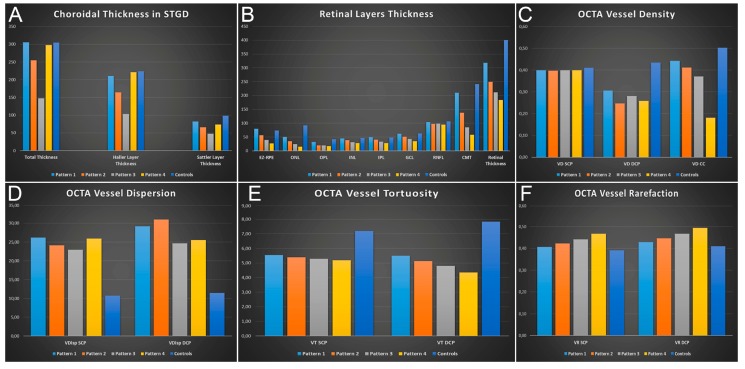
Bar graphs showing all the quantitative analyses performed at baseline. In particular, the parameters for each choroidal pattern are shown as follows: (**A**) choroidal thickness; (**B**) thickness of each retinal layer; (**C**) vessel density of superficial (SCP), deep (DCP) and choriocapillary (CC) vascular plexa; (**D**) vessel dispersion of SCP, DCP and CC vascular plexa; (**E**) vessel tortuosity of SCP, DCP and CC vascular plexa; (**F**) vessel rarefaction of SCP, DCP and CC vascular plexa. All the parameters and the *p* values are extensively reported in Table S1 and Table S2 of the Supplementary Materials.

Choroidal patterns, evaluated by means of structural OCT, resulted in the following distributions (number of eyes; %): Pattern 1 (15; 15%), Pattern 2 (29; 29%), Pattern 3 (26; 26%), Pattern 4 (30; 30%) (Figure 1). Interestingly, different choroidal patterns between the right and the left eye were found in 19 patients (38%). Choroidal patterns showed statistically significant correlations with BCVA, structural OCT and OCTA parameters. In particular, the higher the choroidal damage (e.g., Pattern 3 or 4), the worse the functional outcome and the anatomical retinal status (Table 2). With respect to the choroidal alterations, Pattern 1 showed no significant changes if compared with controls considering the entire choroidal thickness and the separate measurement of Sattler and Haller layers, whereas Pattern 2 and 3 turned out to be statistically significantly thinner with respect to the control eyes (Pattern 3 resulted in the thinnest choroidal pattern when compared to the other STGD subgroups and to controls). On the other hand, Pattern 4 disclosed a significant thinning of the CT and Sattler layer, but not of the Haller layer, with respect to the controls. The post-hoc results are shown in Figure 2 and all the values are extensively reported in Table S1. Complete post-hoc subgroups analysis of clinical and imaging data is also shown in Figure 2; all the parameters and *p* values are extensively reported in Table S2. In particular, no significant age differences were detected in our STGD subgroups. The four choroidal patterns showed gradually worse BCVA, retinal layers thinning and higher HF numbers from Pattern 1 to Pattern 4. With respect to OCTA quantitative analysis, SCP did not show remarkable alterations between STGD groups and healthy controls in terms of VD and VDisp, whereas VT and VR showed progressive worsening from Pattern 1 to Pattern 4. Moreover, the DCP and CC results already strongly affected our patients’ subgroups, also in this case showing an incremental trend of alterations from pattern 1 to 4.

**Figure 3:**
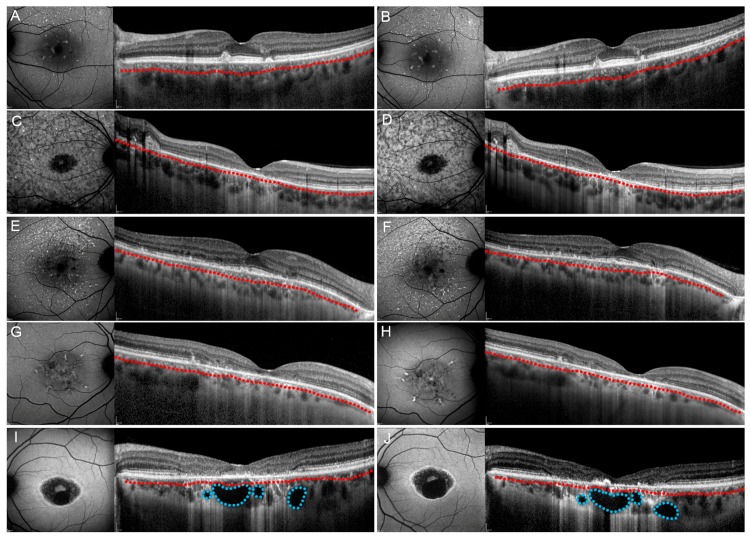
Stargardt disease progression after one year. Fundus autofluorescence-FAF is shown on the left and structural optical coherence tomography-OCT is shown on the right parts of each image. Pattern 1 (**A**,**B**), Pattern 2 (reduced Sattler layer) (**C**,**D**) and Pattern 2 (reduced Haller layer) (**E**,**F**) showed unremarkable changes both on FAF and structural OCT after one year, followed by unremarkable changes of best corrected visual acuity (BCVA). On the contrary, Pattern 3 (**G**,**H**) and Pattern 4 (**I**,**J**) showed both imaging and BCVA worsening after one year. In all images, Sattler and Haller layers are separated by a red dotted line. Moreover, choroidal cavers are highlighted by blue dotted lines.

**Figure 4:**
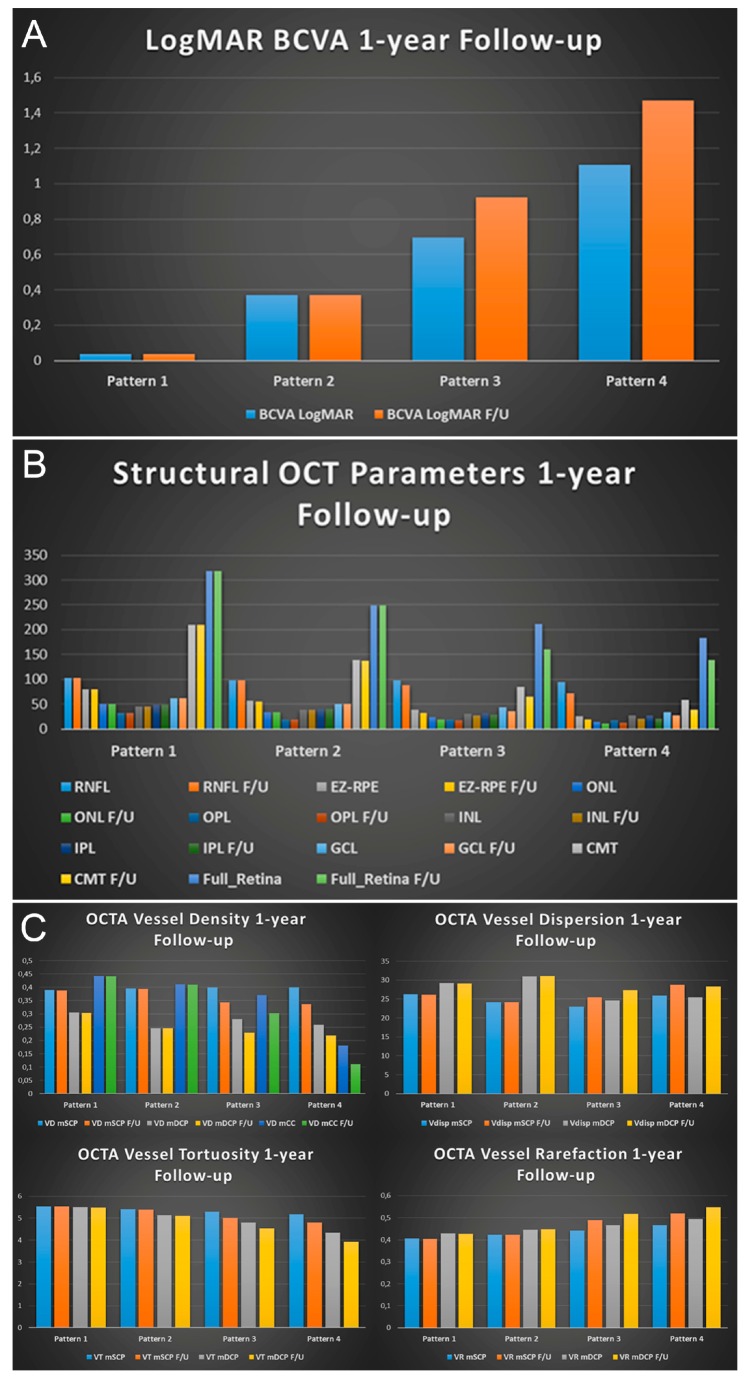
Bar graphs showing all the quantitative changes disclosed after 1-year of follow-up for each choroidal pattern. In details, the parameters for each choroidal pattern are shown as follows: (**A**) LogMAR BCVA; (**B**) thickness of each retinal layer, central macular thickness (CMT and retinal thickness; (**C**) quantitative OCTA parameters, namely vessel density, vessel dispersion, vessel tortuosity and vessel rarefaction. All the parameters and the p values are extensively reported in Table S3 of the Supplementary Materials.

After one year of follow-up, our patients’ subgroups showed different progression features. All patients maintained the same choroidal pattern found at baseline. Pattern 1 and Pattern 2 showed unremarkable functional and anatomical changes (all *p* > 0.05) (Figure 3). Pattern 3 and Pattern 4 revealed significant structural and functional deterioration over the one-year follow-up (Figure 3). Overall, BCVA tended to decrease over the one-year follow-up, together with the worsening of structural OCT and OCTA parameters. All the quantitative parameters are shown in Figure 4. Moreover, all values are extensively reported in Table S3.

## 4. Discussion

Several previous reports showed a strong impairment of the choriocapillaris/choroidal complex in STGD. In detail, CC turned out to be broadly damaged, and significant choroidal changes defined as thinning and reduction of the vascular component were described [5,6,7]. However, no study has specifically focused on the qualitative and quantitative investigation of choroidal changes and on their relationship with clinical outcomes.

In the present study, we divided our STGD cohort of patients in accordance with choroidal structural OCT features in four different subgroups: Pattern 1 (normal appearing choroid), Pattern 2 (damaged Sattler or Haller layer), Pattern 3 (damaged Sattler and Haller layers), and Pattern 4 (damaged Sattler and/or Haller layers with choroidal caverns). Remarkably, approximatively 40% of patients showed choroidal pattern discordance between the right and the left eye, confirming the high phenotypic heterogeneity of STGD manifestation. Choroidal patterns showed a strong relationship both with anatomical and functional retinal status. In particular, we could differentiate Pattern 1, with a milder disease phenotype, and Pattern 4 was characterized by severe anatomical and visual alterations. Moreover, our classification allowed us to determine the variable progression of the disease. Pattern 1 and Pattern 2 did not show remarkable signs of progression after one year of follow-up. On the contrary, Pattern 3 and Pattern 4 disclosed an evident worsening of severity of the disease.

As the choroidal condition is responsible for the perfusion of the CC, and thus of the outer retina, variable degrees of choroidal impairment may lead to different disease severity. This might be the reason why we were able to distinguish four clinically and anatomically different patients’ subgroups as well as to identify different signs of progression at the one-year follow-up. Similar observations can be applied when looking at the behavior of HF in our subgroups; indeed, Pattern 3 and 4 showed higher numbers of HF and these correlated with worse anatomical and visual outcomes, as already reported [8,9].

The interpretation of our findings is challenging. After the initial photoreceptor-RPE genetic-induced impairment, the down-regulation and reduced release of cytokines and growth factors (e.g., VEGF) takes place, leading to cellular and vascular damage both at retinal and choroidal levels [10,11]. Thus, the resulting vascular impairment, although a secondary event in STGD pathogenesis, might proceed as a distinct factor, potentially affecting the progression of the disease. Nevertheless, we cannot exclude that the four patterns represent different stages of STGD progression. Just a longitudinal follow-up of affected patients could elucidate the role and the involvement of the choroid in the pathogenesis of STGD.

We are aware that our study had several limitations. First of all, OCTA analysis depends on high-quality images that can be affected by a number of artefacts potentially influencing the results [12,13]. From this point of view, we adopted all the possible precautions to minimize the potential effect of these artefacts on data analysis. Furthermore, we acknowledge that our quantitative imaging-based analysis might certainly benefit from histopathological validation that is currently lacking. Lastly, the number of cases and the duration of the follow-up were still too limited in order to draw definitive conclusions.

In conclusion, our study identified four choroidal patterns in STGD, which were significantly related to the choroidal-retinal status and visual function of our patients. Moreover, choroidal patterns classification may provide useful information in terms of disease progression, thus, showing a potential role in future interventional clinical trials and, more extensively, in clinical practice. Indeed, in the future perspective of potential drugs interfering with the natural clinical history of STGD, accurate patients’ selection and categorization may help in evaluating treatments’ effects and an achievable outcome. Furthermore, larger prospective studies are warranted to better assess the role of the choroid in STGD

## Figures and Tables

**Table 1:** Clinical and imaging findings.

Parameter	STGD Patients		Controls		*p* Value
	MEAN	STD	MEAN	STD	
BCVA	0.63	0.44	0.00	0.00	All *p* < 0.01
RNFL	97.87	8.79	106.53	9.63	
EZ-RPE	46.36	21.84	73.01	5.55	
ONL	27.94	14.47	92.34	7.25	
OPL	20.57	10.10	41.51	5.98	
INL	34.18	8.09	46.17	6.46	
IPL	35.89	9.08	48.21	7.12	
GCL	45.54	11.24	62.49	7.48	
CMT	111.09	61.35	241.03	11.16	
Retinal Thickness	230.27	54.98	400.66	19.06	
Choroidal Thickness	246.87	83.44	322.53	44.37	
HF_retina	7.05	6.98	0.00	0.00	
HF_choroid	17.08	17.49	0.00	0.00	
VD SCP	0.40	0.02	0.41	0.01	
VD DCP	0.27	0.06	0.43	0.01	
VD CC	0.34	0.15	0.50	0.01	
VDisp SCP	24.69	8.20	10.72	4.15	
VDisp DCP	27.43	9.15	11.45	3.48	
VT SCP	5.33	0.15	7.20	0.31	
VT DCP	4.86	0.50	7.84	0.34	
VR SCP	0.44	0.03	0.39	0.01	
VR DCP	0.46	0.03	0.41	0.01	

**Table 2:** Correlation Analysis.

		BCVA LogMAR	RNFL	EZ-RPE	ONL	OPL	INL	IPL	GCL	CMT	Retinal Thickness	HF_retina	HF_choroid	VD CC	VT SCP	VT DCP	VR SCP	VR DCP
CHOROIDAL PATTERN	Tau Kendall Coeff.	0.793	−0.259	−0.713	−0.712	−0.219	−0.707	−0.708	−0.716	−0.711	−0.709	0.551	0.609	−0.671	−0.707	−0.71	0.691	0.703
	*p* Value	<0.01	<0.01	<0.01	<0.01	<0.01	<0.01	<0.01	<0.01	<0.01	<0.01	<0.01	<0.01	<0.01	<0.01	<0.01	<0.01	<0.01

## Supplementary Materials

The following are available online at https://www.mdpi.com/2077-0383/8/9/1388/s1, Table S1: Choroidal thickness in STGD, Table S2: STGD subgroups analysis, Table S3: Functional and Morphological Changes of STGD patients after 1-year of Follow-up.
