# Supplementary material for: Choroidal Patterns in Stargardt Disease: Correlations with Visual Acuity and Disease Progression
Source: J Clin Med. 2019 Sep 5;8(9):1388. doi: 10.3390/jcm8091388 (PMC6780607; doi:10.3390/jcm8091388)
Supplement: Supplementary file 1 [file jcm-08-01388-s001.pdf]

Supplementary Materials

Table S1: Choroidal thickness in STGD.

Choroidal Thickness in STGD															
**Choroidal Total Thickness**															
	**Pattern 1**	**Pattern 2**	**Pattern 3**	**Pattern 4**	**Controls**	**Control vs P1**	**Control vs P2**	**Control vs P3**	**Control vs P4**	**P1 vs P2**	**P1 vs P3**	**P1 vs P4**	**P2 vs P3**	**P2 vs P4**	**P3 vs P4**
**Mean**	305	255	147	298	305	p>0,05	p<0.01	p<0.01	p<0.01	p<0.01	p<0.01	p>0,05	p<0.01	p>0,05	p<0.01
**STD**	39	50	77	15	59										
**Choroidal Haller Layer Thickness**															
	Pattern 1	Pattern 2	Pattern 3	Pattern 4	Controls	Control vs P1	Control vs P2	Control vs P3	Control vs P4	P1 vs P2	P1 vs P3	P1 vs P4	P2 vs P3	P2 vs P4	P3 vs P4
**Mean**	211	164	103	221	224	p>0,05	p<0.01	p<0.01	p>0,05	p<0.01	p<0.01	p>0,05	p<0.01	p<0.01	p<0.01
**STD**	25	32	54	17	48										
**Choroidal Sattler Layer Thickness**															
	Pattern 1	Pattern 2	Pattern 3	Pattern 4	Controls	Control vs P1	Control vs P2	Control vs P3	Control vs P4	P1 vs P2	P1 vs P3	P1 vs P4	P2 vs P3	P2 vs P4	P3 vs P4
**Mean**	82	66	48	74	98	p>0.05	p<0.01	p<0.01	p<0.01	p<0.01	p<0.01	p>0.05	p<0.01	p>0.05	p<0.01
**STD**	14	8	37	21	27										

Table S2: STGD subgroups analysis.

STGD Subgroups Analysis													
**Parameter**	**Choroidal Pattern**	**MEAN**	**STD**	**p values**									
				1 vs 2	1 vs 3	1 vs 4	1 vs Controls	2 vs 3	2 vs 4	2 vs Controls	3 vs 4	3 vs Controls	4 vs Controls
AGE	1	49	7	>0,05	>0,05	>0,05	>0,05	>0,05	>0,05	>0,05	>0,05	>0,05	>0,05
	2	44	15										
	3	38	15										
	4	44	18										
	Controls	45	18										
BCVA (logMAR)	1	0,04	0,08	<0,01	<0,01	<0,01	>0,05	<0,01	<0,01	<0,01	<0,01	<0,01	<0,01
	2	0,37	0,20										
	3	0,69	0,22										
	4	1,11	0,30										
	Controls	0,0	0,0										
EZ-RPE	1	80	12	<0,01	<0,01	<0,01	>0,05	<0,01	<0,01	<0,01	<0,01	<0,01	<0,01
	2	56	15										

	3	39	11										
	4	26	10										
	Controls	73	6										
	1	50	9										
	2	34	10										
ONL	3	24	7	<0,01	<0,01	<0,01	<0,01	<0,01	<0,01	<0,01	<0,01	<0,01	<0,01
	4	15	7										
	Controls	92	7										
	1	32	14										
	2	20	11										
OPL	3	19	6	<0,01	<0,01	<0,01	<0,01	>0,05	>0,05	<0,01	>0,05	<0,01	<0,01
	4	17	6										
	Controls	42	6										
	1	45	6										
	2	38	6										
INL	3	31	5	<0,01	<0,01	<0,01	>0,05	<0,01	<0,01	<0,01	>0,05	<0,01	<0,01
	4	27	4										
	Controls	46	6										
	1	49	7										
	2	40	6										
IPL	3	33	4	<0,01	<0,01	<0,01	>0,05	<0,01	<0,01	<0,01	>0,05	<0,01	<0,01
	4	28	4										
	Controls	48	7										
	1	62	4										
	2	51	6										
GCL	3	43	6	<0,01	<0,01	<0,01	>0,05	<0,01	<0,01	<0,01	<0,01	<0,01	<0,01
	4	35	8										
	Controls	62	7										
	1	104	5										
	2	98	8										
RNFL	3	98	11	>0,05	>0,05	>0,05	>0,05	>0,05	>0,05	<0,01	>0,05	<0,01	<0,01
	4	95	8										
	Controls	107	10										
	1	209	25										
	2	138	47										
CMT	3	85	31	<0,01	<0,01	<0,01	<0,01	<0,01	<0,01	<0,01	>0,05	<0,01	<0,01
	4	58	17										
	Controls	241	11										
	1	318	44										
	2	249	39										
Retinal Thickness	3	212	22	<0,01	<0,01	<0,01	<0,01	<0,01	<0,01	<0,01	<0,01	<0,01	<0,01
	4	184	24										
	Controls	401	19										
	1	0	1										
HF Retina	2	4	4	>0,05	<0,01	<0,01	>0,05	<0,01	<0,01	>0,05	>0,05	<0,01	<0,01
	3	11	7										

	4	10	7										
	Controls	0	0										
	1	1	1										
	2	10	8										
HF Choroid	3	21	17	>0,05	<0,01	<0,01	>0,05	<0,01	<0,01	>0,05	>0,05	<0,01	<0,01
	4	28	20										
	Controls	0	0										
	1	0,40	0,01										
	2	0,40	0,02										
VD SCP	3	0,40	0,02	>0,05	>0,05	>0,05	>0,05	>0,05	>0,05	>0,05	>0,05	>0,05	>0,05
	4	0,40	0,01										
	Controls	0,41	0,01										
	1	0,31	0,01										
	2	0,25	0,07										
VD DCP	3	0,28	0,05	>0,05	>0,05	>0,05	<0,01	>0,05	>0,05	<0,01	>0,05	<0,01	>0,05
	4	0,26	0,06										
	Controls	0,43	0,01										
	1	0,44	0,01										
	2	0,41	0,07										
VD CC	3	0,37	0,06	<0,01	<0,01	<0,01	<0,01	<0,01	<0,01	<0,01	<0,01	<0,01	<0,01
	4	0,18	0,18										
	Controls	0,50	0,01										
	1	26,23	5,87										
	2	24,12	5,70										
VDisp SCP	3	22,94	7,11	>0,05	>0,05	>0,05	<0,01	>0,05	>0,05	<0,01	>0,05	<0,01	>0,05
	4	25,95	11,58										
	Controls	10,72	4,15										
	1	29,21	10,53										
	2	30,97	10,34										
VDisp DCP	3	24,66	8,13	>0,05	>0,05	>0,05	<0,01	>0,05	>0,05	<0,01	>0,05	<0,01	>0,05
	4	25,54	7,21										
	Controls	11,45	3,48										
	1	5,54	0,08										
	2	5,40	0,10										
VT SCP	3	5,30	0,07	>0,05	<0,01	<0,01	<0,01	>0,05	<0,01	<0,01	>0,05	<0,01	<0,01
	4	5,19	0,10										
	Controls	7,20	0,31										
	1	5,50	0,16										
	2	5,13	0,30										
VT DCP	3	4,80	0,23	<0,01	<0,01	<0,01	<0,01	<0,01	<0,01	<0,01	<0,01	<0,01	<0,01
	4	4,35	0,41										
	Controls	7,84	0,34										
	1	0,41	0,01										
VR SCP	2	0,42	0,02	>0,05	<0,01	<0,01	>0,05	<0,01	<0,01	<0,01	<0,01	<0,01	<0,01
	3	0,44	0,01										
	4	0,47	0,03										

	Controls	0,39	0,01										
	1	0,43	0,01										
	2	0,45	0,02										
VR DCP	3	0,47	0,01	<0,01	<0,01	<0,01	<0,01	<0,01	<0,01	<0,01	<0,01	<0,01	<0,01
	4	0,49	0,03										
	Controls	0,41	0,01										

Table S3: Functional and Morphological Changes of STGD patients after 1-year of Follow-up.

**Functional and Morphological Changes of STGD patients after 1-year of Follow-up**													
**Parameter**	**Visit**	**Choroidal Pattern 1**			**Choroidal Pattern 2**			**Choroidal Pattern 3**			**Choroidal Pattern 4**		
		**MEAN**	**STD**	**p value**	**MEAN**	**STD**	**p value**	**MEAN**	**STD**	**p value**	**MEAN**	**STD**	**p value**
**BCVA LogMAR**	Baseline	0,04	0,08	p>0.05	0,37	0,20	p>0.05	0,69	0,22	p<0.05	1,11	0,30	p<0.01
	1-year Follow-up	0,04	0,08		0,37	0,20		0,92	0,32		1,47	0,38	
**Choroidal Thickness**	Baseline	305,55	38,86	p>0.05	255,20	50,07	p>0.05	147,40	76,74	p<0.05	297,56	14,61	p<0.01
	1-year Follow-up	302,41	38,41		247,47	48,61		94,13	48,58		160,84	7,90	
**Sattler Layer Thickness**	Baseline	82,17	13,65	p>0.05	65,80	8,29	p>0.05	48,00	37,49	p>0.05	73,50	21,43	p<0.01
	1-year Follow-up	81,27	13,51		63,82	8,02		30,41	23,61		39,73	11,58	
**Haller Layer Thickness**	Baseline	210,58	25,29	p>0.05	164,00	32,38	p>0.05	108,80	53,98	p<0.05	221,33	17,25	p<0.01
	1-year Follow-up	208,43	25,05		159,02	31,39		69,49	34,21		119,64	9,32	
**RNFL**	Baseline	103,66	4,99	p>0.05	97,82	7,72	p>0.05	98,47	10,72	p<0.01	94,50	8,33	p<0.01
	1-year Follow-up	103,38	4,98		97,45	7,69		87,46	9,46		71,41	6,15	
**EZ-RPE**	Baseline	79,54	11,93	p>0.05	56,24	14,97	p>0.05	39,47	11,06	p<0.05	26,25	10,20	p<0.05
	1-year Follow-up	79,33	11,90		56,03	14,92		32,50	8,62		19,86	7,68	
**ONL**	Baseline	49,71	8,87	p>0.05	34,48	10,09	p>0.05	23,55	6,59	p<0.05	14,60	7,05	p<0.05
	1-year Follow-up	49,58	8,84		34,34	10,04		19,34	5,28		10,19	5,09	
**OPL**	Baseline	31,68	13,74	p>0.05	19,52	10,91	p>0.05	19,48	6,36	p>0.05	16,92	5,64	p<0.01
	1-year Follow-up	31,60	13,71		19,45	10,86		17,29	5,66		12,79	4,29	
**INL**	Baseline	45,05	5,62	p>0.05	38,44	6,09	p>0.05	31,36	4,52	p<0.05	27,12	4,07	p<0.01
	1-year Follow-up	44,93	5,60		38,29	6,07		27,84	4,10		20,65	3,17	
**IPL**	Baseline	49,18	6,67	p>0.05	40,17	6,39	p>0.05	32,91	4,34	p<0.01	27,72	4,13	p<0.01
	1-year Follow-up	49,05	6,65		40,02	6,36		29,22	3,74		21,23	3,37	
**GCL**	Baseline	61,52	3,60	p>0.05	50,83	6,31	p>0.05	43,12	5,69	p<0.01	34,57	8,16	p<0.01
	1-year Follow-up	61,36	3,60		50,64	6,29		35,68	5,11		27,65	5,63	
**CMT**	Baseline	209,38	25,25	p>0.05	138,24	46,86	p>0.05	85,20	31,37	p<0.05	58,17	17,20	p<0.01
	1-year Follow-up	208,82	25,19		137,72	46,67		64,45	23,90		38,14	11,31	
**Retinal Thickness**	Baseline	318,29	44,30	p>0.05	249,35	38,52	p>0.05	211,74	22,45	p<0.01	183,88	23,79	p<0.01
	1-year Follow-up	317,45	44,18		248,42	38,39		160,00	17,03		139,08	17,98	
**VD SCP**	Baseline	0,39	0,01	p>0.05	0,40	0,02	p>0.05	0,40	0,02	p<0.01	0,40	0,01	p<0.01
	1-year Follow-up	0,39	0,01		0,39	0,02		0,34	0,02		0,34	0,02	
**VD DCP**	Baseline	0,31	0,01	p>0.05	0,25	0,07	p>0.05	0,28	0,05	p<0.01	0,26	0,06	p<0.05
	1-year Follow-up	0,30	0,01		0,25	0,07		0,23	0,04		0,22	0,05	
**VD CC**	Baseline	0,44	0,01	p>0.05	0,41	0,07	p>0.05	0,37	0,06	p<0.01	0,18	0,18	p>0.05
	1-year Follow-up	0,44	0,01		0,41	0,07		0,30	0,05		0,11	0,11	
**VDisp SCP**	Baseline	26,23	5,87	p>0.05	24,12	5,70	p>0.05	22,94	7,11	p>0.05	25,95	11,58	p>0.05
	1-year Follow-up	26,16	5,86		24,21	5,72		25,46	7,92		28,81	12,89	
**VDisp DCP**	Baseline	29,21	10,53	p>0.05	30,97	10,34	p>0.05	24,66	8,13	p>0.05	25,54	7,21	p>0.05
	1-year Follow-up	29,14	10,50		31,08	10,38		27,35	9,00		28,28	7,96	
**VT SCP**	Baseline	5,54	0,08	p>0.05	5,40	0,10	p>0.05	5,30	0,07	p<0.01	5,19	0,10	p<0.01
	1-year Follow-up	5,53	0,08		5,38	0,10		5,00	0,10		4,81	0,09	
**VT DCP**	Baseline	5,50	0,16	p>0.05	5,13	0,30	p>0.05	4,80	0,23	p<0.01	4,35	0,41	p<0.01

	1-year Follow-up	5,49	0,16		5,11	0,30		4,53	0,22		3,93	0,37	
**VR SCP**	Baseline	0,41	0,01	p>0.05	0,42	0,02	p>0.05	0,44	0,01	p<0.01	0,47	0,03	p<0.01
	1-year Follow-up	0,41	0,01		0,42	0,02		0,49	0,01		0,52	0,03	
**VR DCP**	Baseline	0,43	0,01	p>0.05	0,45	0,02	p>0.05	0,47	0,01	p<0.01	0,49	0,03	p<0.01
	1-year Follow-up	0,43	0,01		0,45	0,02		0,52	0,02		0,55	0,03	
